# Dr. Edward J. Cook

**Published:** 1917-07

**Authors:** 


**Dr. Edward J. Cook,** Niagara 1895, of Buffalo, died at his home, June 8, aged 74. He was born in Clarendon, N. Y., and was educated at the Rochester City University. In early life, he was a Methodist clergyman, serving as circuit minister in western N. Y., where he established 12 churches, among them one at Holley, of which he was pastor for a number of years. He began the study of medicine in 1891 and, after graduating, practiced in Buffalo. His only son is Dr. Bruce L. D. Cook of Buffalo.

